# Exhausted Cytotoxic Control of Epstein-Barr Virus in Human Lupus

**DOI:** 10.1371/journal.ppat.1002328

**Published:** 2011-10-20

**Authors:** Martin Larsen, Delphine Sauce, Claire Deback, Laurent Arnaud, Alexis Mathian, Makoto Miyara, David Boutolleau, Christophe Parizot, Karim Dorgham, Laura Papagno, Victor Appay, Zahir Amoura, Guy Gorochov

**Affiliations:** 1 Institut National de la Santé et de la Recherche Médicale (Inserm) UMR-S 945, Paris, France; 2 UPMC Université Paris 06, Paris, France; 3 Laboratoire AP-HP de Virologie, C.H.U. Pitié-Salpêtrière, Paris, France; 4 Service de Médecine Interne 2, Centre National de Référence des Lupus et Syndrome des Antiphospholipides, C.H.U. Pitié-Salpêtrière, Paris, France; 5 Laboratoire AP-HP d'Immunologie Cellulaire et Tissulaire, Paris, France; NIH/NIAID, United States of America

## Abstract

Systemic Lupus Erythematosus (SLE) pathology has long been associated with an increased Epstein-Barr Virus (EBV) seropositivity, viremia and cross-reactive serum antibodies specific for both virus and self. It has therefore been postulated that EBV triggers SLE immunopathology, although the mechanism remains elusive. Here, we investigate whether frequent peaks of EBV viral load in SLE patients are a consequence of dysfunctional anti-EBV CD8^+^ T cell responses. Both inactive and active SLE patients (n = 76 and 42, respectively), have significantly elevated EBV viral loads (*P* = 0.003 and 0.002, respectively) compared to age- and sex-matched healthy controls (n = 29). Interestingly, less EBV-specific CD8^+^ T cells are able to secrete multiple cytokines (IFN-γ, TNF-α, IL-2 and MIP-1β) in inactive and active SLE patients compared to controls (*P* = 0.0003 and 0.0084, respectively). Moreover, EBV-specific CD8^+^ T cells are also less cytotoxic in SLE patients than in controls (CD107a expression: *P* = 0.0009, Granzyme B release: *P* = 0.0001). Importantly, cytomegalovirus (CMV)-specific responses were not found significantly altered in SLE patients. Furthermore, we demonstrate that EBV-specific CD8^+^ T cell impairment is a consequence of their Programmed Death 1 (PD-1) receptor up-regulation, as blocking this pathway reverses the dysfunctional phenotype. Finally, prospective monitoring of lupus patients revealed that disease flares precede EBV reactivation. In conclusion, EBV-specific CD8^+^ T cell responses in SLE patients are functionally impaired, but EBV reactivation appears to be an aggravating consequence rather than a cause of SLE immunopathology. We therefore propose that autoimmune B cell activation during flares drives frequent EBV reactivation, which contributes in a vicious circle to the perpetuation of immune activation in SLE patients.

## Author Summary

Systemic Lupus Erythematosus (SLE) has been associated with Epstein-Barr Virus (EBV) infection for decades, however the mechanistic links have remained elusive. Most human adults are infected by EBV and carry the virus for life without clinical symptoms. However, for unknown reasons EBV induces infectious mononucleosis in some individuals, during which cross-reactive antibodies specific for both virus and self have been detected. Interestingly, such cross-reactive antibodies are also frequently found in SLE patients. Since, EBV seropositivity and viremia are more frequent in SLE patients than in healthy individuals, it has been postulated that EBV trigger autoimmunity. Here we show that SLE patients are indeed less capable of controlling EBV viremia, since their EBV-specific CD8^+^ T cells have diminished capacity to secrete effector molecules (e.g. cytokines and chemokines) and to kill EBV-infected targets as a consequence of their Programmed Death 1 (PD-1) receptor up-regulation. Longitudinal studies further reveal that disease flares precede EBV viremia. Thus, contrary to expectations, EBV reactivation appears to be an aggravating consequence, rather than a cause, of SLE immunopathology. Our results pave the way for immunological interventions that restore the host-EBV balance, which may result in decreased levels of aggravating cross-reactive antibodies and ultimately be beneficial to SLE patients.

## Introduction

Systemic lupus erythematosus (SLE) is a chronic autoimmune disorder. Common manifestations include inflammation and tissue damage of skin and joints as well as inner organs, such as brain and kidneys, in severe cases. The disease can be fatal, but with recent medical advances, mortality is reduced significantly. The course of the disease is unpredictable, with peak periods of illness (active SLE) alternating with periods of remission (inactive SLE).

SLE-related autoimmune symptoms can be triggered by environmental factors, such as ultraviolet light, drugs and viruses.[1], [2] In this regard, it has been reported that lupus patients have elevated antibody responses to the gamma-herpesvirus EBV [3], [4] and that this antibody response shows cross-reactivity to nuclear self antigens.[5], [6], [7], [8] Primary EBV infection typically occurs during childhood without apparent clinical symptoms and evolves into a non-symptomatic life-long virus carrying latency. Rare cases of infection in early adulthood lead to infectious mononucleosis (IM), which has been linked to increased risk of Hodgkin's lymphoma [9] and to the onset of autoimmune diseases, such as Multiple Sclerosis (MS) [10] and less documented cases of rheumatoid arthritis (RA) and SLE, as reviewed by Münz *et al.* [2] Detectable levels of lytic EBV antigen, BZLF1, were observed more frequently in SLE patients (35%) than in healthy controls (0%), suggesting recurrent EBV replication in SLE patients.[11] In line with this observation, several groups demonstrated that EBV viral load is elevated in SLE patients,[12], [13] and that the number of infected B cells monitored longitudinally is positively correlated with the SLE disease activity index (SLEDAI).[11] However, the mechanisms linking EBV to SLE immunopathology still remain elusive. On the one hand, EBV-related disorders are often observed as a consequence of immunodeficiency in hosts, such as bone marrow transplant patients.[14] On the other hand, it is debated that EBV transformation can support the survival of self-reactive B cells.[2] It has furthermore been demonstrated that EBV nuclear antigen 1 (EBNA1) is capable of inducing T [15], [16] and B cell responses [5], [6], [7], [8] cross-reactive to auto-antigens, and thus potentially induce auto-immunity. Of note, IM patients have cross-reactive antibody responses to EBNA1 and the common lupus spliceosomal autoantigen Sm B' during the most severe acute phase of IM,[17] suggesting a connection between the immunopathology of EBV-induced IM and SLE.[18]

It was reported in an early study that T cells from SLE patients are unable to control immunoglobulin production from EBV-exposed B cells.[19] Subsequently, Kang *et al.* observed that lupus patients had elevated frequencies of interferon-γ (IFN-γ) secreting EBV-specific CD4^+^ T cells, whereas no significant modification was observed for IFN-γ secreting EBV-specific CD8^+^ T cells.[12] Similarly, Berner *et al.* reported that the frequency of EBV-specific CD8^+^ T cells did not differ between SLE patients and healthy controls, when analysed using peptide-MHC tetramer probes. However, the capacity of EBV-specific CD8^+^ T cells to secrete IFN-γ seemed reduced in SLE patients compared to healthy controls.[20] Altogether, whether the defective control of latent EBV infection in SLE patients is related to a CD8^+^ T cell defect remains controversial.[11], [12], [13] Furthermore, it is unclear whether the defect is EBV-specific or global. Finally, the sequence in which EBV re-activation and disease onset occurs is unresolved.

Here, we assess quantitative and qualitative attributes of EBV-specific CD8^+^ T cells from SLE patients. We show that the frequencies of IFN-γ, tumour necrosis factor-α (TNF-α), interleukin-2 (IL-2) and Macrophage Inflammatory Protein 1β (MIP-1β or CCL4) secretion by EBV-specific CD8^+^ T cells upon antigen stimulation are diminished in SLE patients compared to healthy controls. We furthermore demonstrate that EBV-specific T cells from SLE patients exhibit a marked impairment in their cytotoxic granule exocytosis process. We finally associate the dysfunctional T cell phenotype with the up-regulation of the inhibitory receptor programmed death 1 (PD-1), and strengthen this association by reversing the dysfunctional T cell phenotype through specific blockade of the PD-1 signaling pathway. In line with previous findings, EBV viral load was found to be elevated in SLE patients compared to healthy controls. Interestingly, longitudinal monitoring revealed that bursts of viral load always occurred in a delayed manner with respect to disease flare onset.

## Results

### SLE patients have elevated EBV viral load

**Figure 1:**
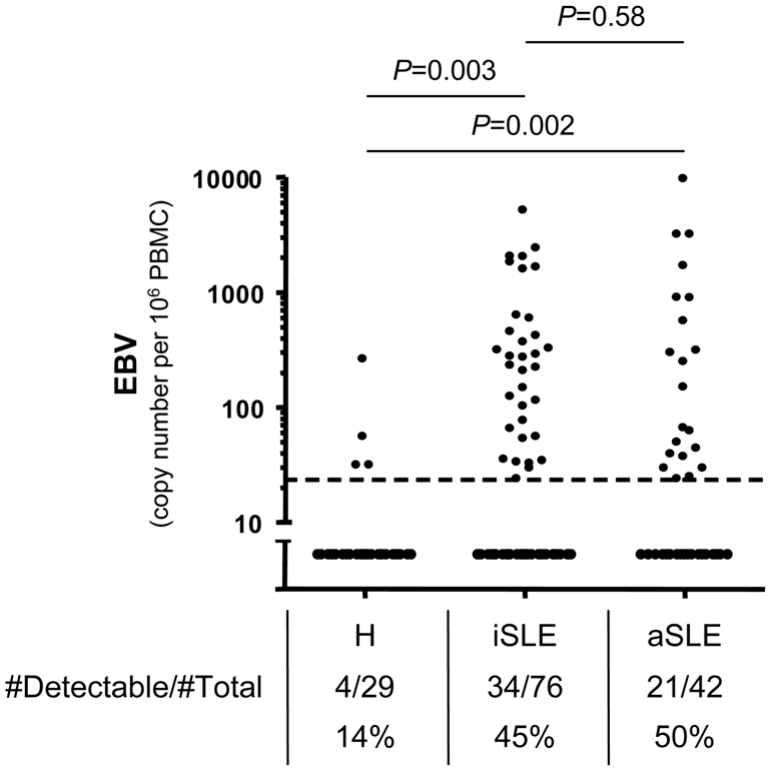
Cell-associated EBV viral load in SLE patients. qPCR measurements of EBV genomes per 10^6^ PBMCs from EBV seropositive healthy controls (H, *n* = 29), inactive (iSLE, *n* = 76) and active (aSLE, *n* = 42) SLE patients. The absolute number and the frequency of individuals having viral loads above the detection limit of 25 viral genomes per 10^6^ PBMCs (dotted line) are indicated. Group comparisons are performed with Fisher's exact test.

To study the impact of EBV infection on SLE immunopathology, we established a cohort of SLE patients and age- and sex-matched healthy controls. Patient characteristics and treatments are presented in Table 1. We validated that the patients displayed the EBV associated features identified in literature,[3], [4] such as increased EBV seroprevalance (*P* = 0.006) and augmented anti-EBV antibody titers (*P*<0.0001) (Table 1). Furthermore, we confirm that cell-associated EBV viral load is augmented in EBV seropositive SLE patients, when compared with EBV seropositive healthy controls.[12], [13] Thus, cell-associated EBV DNA is more frequently above detection threshold in SLE patients than in healthy controls (Figure 1). In comparison, CMV was below detection threshold in the majority of study subjects (Healthy: 0 of 18; SLE: 5 of 93, *P* = 0.59). We then explored whether cell-associated EBV viral load is linked with disease activity. As shown, EBV was as frequently detectable in inactive as in active patients (Figure 1). EBV viral loads were not influenced by any treatment-related parameters (corticosteroids, hydroxychloroquine and other immunosuppressors – see Table 1) according to a multivariate analysis (*P* = 0.40, 0.21 and 0.24, respectively, *n* = 118).

**Table 1:** Cohort characteristics.

	Healthy controls (*n* = 31)	Inactive SLE patients (*n* = 76)	Active SLE patients (*n* = 42)	*P*-value
Female Sex (%)	27 (87%)	71 (93%)	36 (86%)	0.32
Inclusion age (years), Median	33.0	34.3	34.6	0.92
[Range]	[19–57]	[16–61]	[16–58]	
SLEDAI, Median	N/A	0	8	0.0001
[Range]		[0–5]	[6]–[23]	
EBV Serology	29 (92%)	76 (100%)	42 (100%)	0.006^A^
CMV Serology	18 (58%)	58 (76%)	35 (83%)	0.035^A^
Anti-EBV IgG titers^B^, Median (RU/ml)	10500	17900	19300	<0.0001^A^
[Range]	[2400–25400]	[2900–28900]	[2800–35400]	
Corticosteroid (%)	N/A	73%	65%	0.49
Median (mg/day) [Range]		5 [0–55]	7.5 [0–60]	
Hydroxychloroquine (%)	N/A	89%	87%	0.76
Other immunosuppressors (%)	N/A	23%	26%	0.81

N/A: not applicable, RU: Relative Units.

A Healthy controls versus all SLE patients.

B Only titers from seropositive individuals are included.

### Expansion of EBV-specific CD8^+^ T cells counterbalanced by lymphopenia

**Figure 2:**
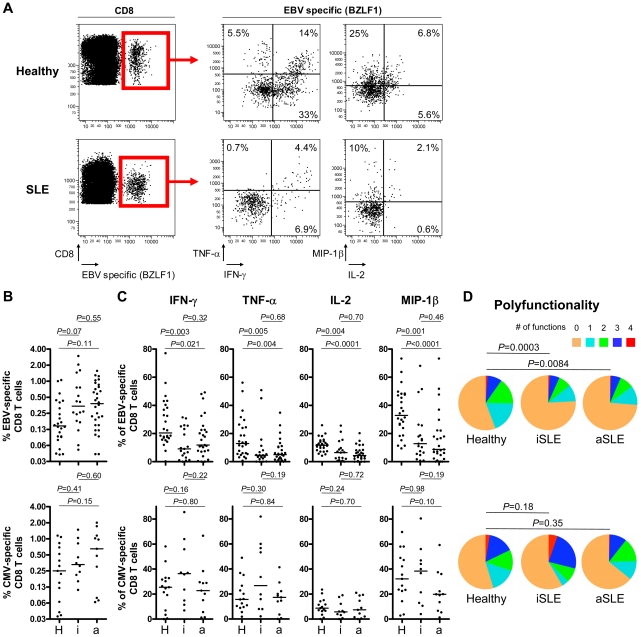
Multiparametric functional assessment of EBV- and CMV-specific CD8^+^ T cells in SLE patients. (**A**) Representative cytofluorometric detection (left) and functional analysis (right) of CD8^+^ T cells specific for one of the lytic EBV antigens tested (BZLF1) in a healthy control (upper panel) and in an inactive SLE patient (lover panel) post peptide antigen stimulation of PBMC. Lytic EBV and CMV antigen-specific cells were detected with peptide/MHC tetramer and anti-CD8 antibody (red box) and simultaneously analyzed for intra-cellular IFN-γ, TNF-α, IL-2 and MIP-1β content. Cytokine/chemokine gates were positioned according to control stains of non-stimulated virus-specific T cells. (B) Magnitude and (C) functionality of EBV- (upper panel) and CMV-specific (lower panel) responses in healthy controls (H, *n* = 26 and 15, respectively), inactive (i, *n* = 19 and 10) and active (a, *n* = 27 and 11) SLE patients. (D) EBV-specific T cells (upper panel) are strikingly less polyfunctional in inactive (iSLE) and active (aSLE) SLE patients compared to controls (healthy), while polyfunctionality of CMV-specific responses (lower panel) is preserved. Pie representations of virus-specific CD8^+^ T cells represent the fraction of individual cells secreting none (0) or any (1, 2, 3 or 4) of the four cytokines IFN-γ, TNF-α, IL-2 and MIP-1β (color coded as indicated). E.g. the red pie slice indicates the proportion of cells producing four cytokines (IFN-γ, TNF-α, IL-2 and MIP-1β). *P*-values monitoring differences between healthy donors and SLE patients are calculated using a non-parametric Mann-Whitney test and pie comparison statistics of the Spice software.

In order to address whether increased EBV viral loads in SLE patients could be due to a T cell functional defect, we compared phenotypic and functional characteristics of lytic (BMLF1, BMRF1, BZLF1) and latent (EBNA3A and EBNA3B) EBV-specific CD8^+^ T cell responses between patients with SLE and healthy controls. Using HLA/peptide tetramers, we quantified circulating lytic and latent EBV- and CMV pp65-specific CD8^+^ T cells in patients and controls (Figure 2A and Figure S1A in Text S1). As shown, inactive and active SLE patients have slightly elevated frequencies of lytic EBV-, and comparable frequencies of latent EBV- and CMV-specific CD8^+^ T cells compared to healthy controls (Figure 2B and Figure S1B in Text S1). However, the elevated lytic EBV-specific CD8^+^ T cell frequency is counterbalanced by a general lymphopenia (Figure S2A in Text S1). Thus, absolute counts of lytic EBV-specific CD8^+^ T cells in SLE patients are comparable (inactive SLE patients) or even slightly decreased (active SLE patients) as compared to healthy controls (Figure S2B in Text S1).

### Defective EBV-specific CD8^+^ T cell cytokine secretion in SLE patients

MHC class I tetramer positive EBV- and CMV-specific CD8^+^ T cells were then tested for their capacity to secrete IFN-γ, TNF-α, IL-2 and MIP-1β in response to stimulation with EBV and CMV cognate antigens (Figure 2A). We found that CD8^+^ T cells from inactive and active SLE patients specific for lytic EBV antigens are functionally impaired in their capacity to secrete IFN-γ (*P* = 0.003 and 0.021, respectively), TNF-α (*P* = 0.005 and 0.004, respectively), IL-2 (*P* = 0.004 and 0.0001, respectively) and MIP-1β (*P* = 0.001 and 0.0001, respectively) compared to T cells from healthy controls (Figure 2C – upper panel). The impairment is also observed as a decline in the absolute number of circulating cytokine-secreting EBV-specific CD8^+^ T cells (Figure S2C in Text S1). Moreover, the proportion of EBV-specific CD8^+^ T cells able to secrete multiple cytokines is reduced in patients compared to controls (Figure 2D – upper panel). Similarly, we observed that CD8^+^ T cells from SLE patients specific for latent EBV antigens tend to have reduced capacity to secrete IFN-γ (Figures S1A and S1C in Text S1). In contrast, CMV-specific cytokine responses are well preserved in inactive and active SLE patients (Figure 2C – lower panel). Likewise, polyfunctionality of CMV-specific CD8^+^ T cells do not differ significantly between patients and controls (Figure 2D – lower panel). Importantly, impaired functionality of EBV-specific CD8^+^ T cells is not related to treatments (corticosteroids, hydroxychloroquine and other immunosupressors) according to a multivariate statistical analysis (All treatment parameters were non-significant for the prediction of IFN-γ-, IL-2-, MIP-1β- and TNF-α-secretion, *n* = 46).

### Impaired EBV-specific cytotoxic granule exocytosis in SLE

**Figure 3:**
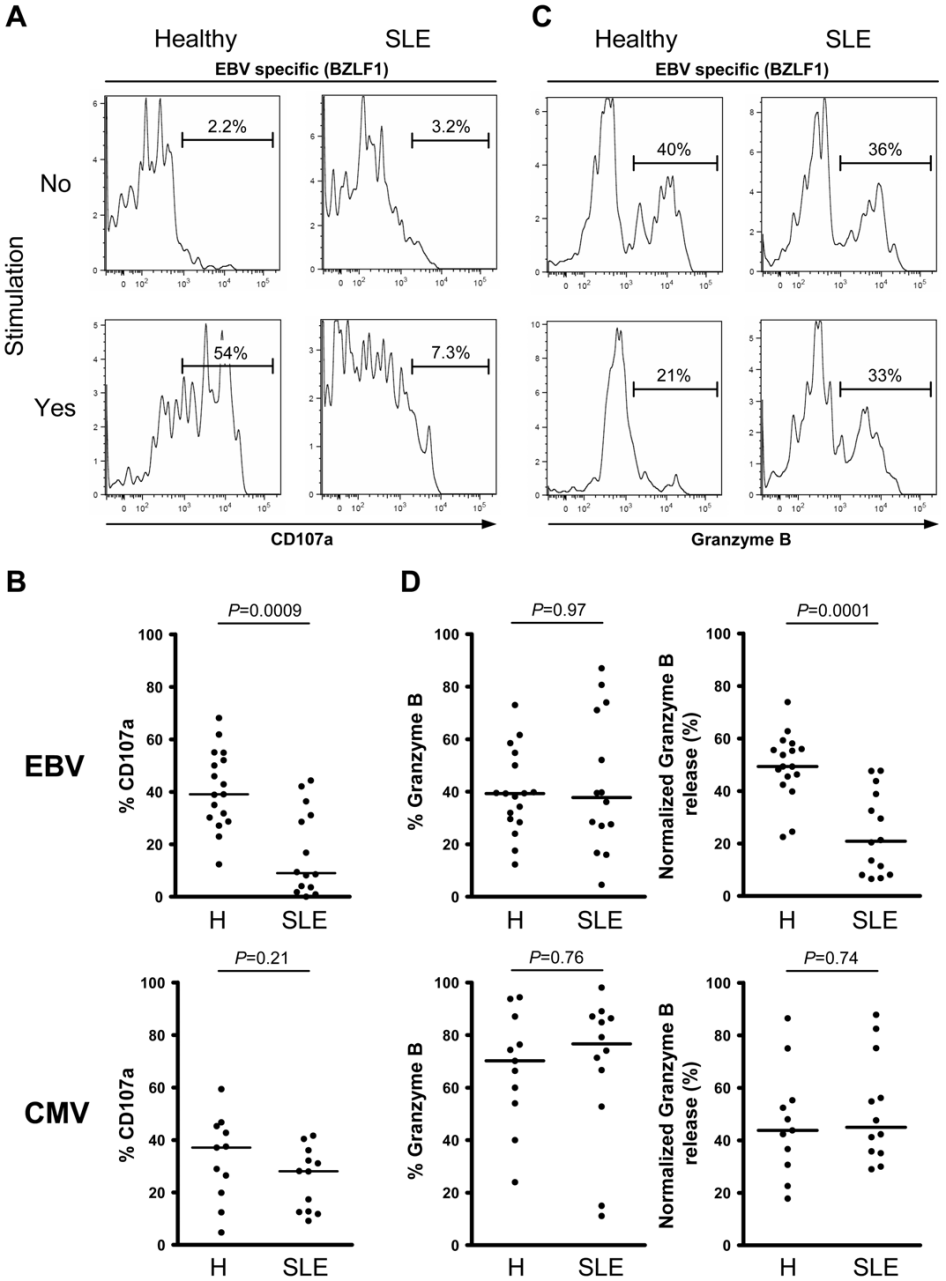
Lytic EBV antigen-specific T cells from SLE patients are impaired in their ability to release their cytotoxic granule content. Representative analysis of (A) CD107a and (C) granzyme B expression in CD8^+^ T cells, specific for one of the lytic EBV antigens tested (BZLF1), from healthy control and SLE patient either *ex vivo* (upper panel) or following cognate antigen stimulation (lower panel). As shown, EBV-specific CD8^+^ T cells from SLE patients are much less able to mobilize surface CD107a and release their granzyme B content upon cognate antigen stimulation. (B) Mobilization of CD107a on the surface of EBV- (upper panel) and CMV-specific (lower panel) CD8^+^ T cells upon cognate antigen stimulation over night. (D) *Ex vivo* analysis of the frequency of granzyme B expression in EBV- and CMV-specific CD8^+^ T cells from healthy controls (*n* = 17 and 11, respectively) and SLE patients (*n* = 14 and 12) (left panel), as well as the frequency of EBV- and CMV- specific CD8^+^ T cells positive for granzyme B capable of releasing their granzyme B upon cognate antigen stimulation (right panel). Healthy controls are compared to SLE patients using a non-parametric Mann-Whitney test.

We then investigated whether EBV-specific CD8^+^ T cells from SLE patients are also less cytotoxic than their healthy counterparts. We measured the capacity of EBV-specific CD8^+^ T cells to degranulate by monitoring the appearance of degranulation marker LAMP-1 (CD107a) on the cell surface (Figures 3A–B) and granzyme B release (Figures 3C–D), prior to and following stimulation with cognate antigen. Surface exposed CD107a is inversely correlated with granzyme B release, and thus a marker of recent history of cytotoxic activity.[21] As shown, CD8^+^ T cells from SLE patients specific for lytic EBV antigens carry similar loads of granzyme B (Figure 3D – upper left panel), but are dramatically less able to degranulate (*P* = 0.0009, Figure 3B upper panel) and release their cytotoxic content (*P* = 0.0001, Figure 3D – upper right panel) following stimulation, compared to EBV-specific CD8^+^ T cells from healthy controls. A similar impairment of cytotoxic activity was observed for CD8^+^ T cells specific for latent EBV antigens (CD107a, *P* = 0.050) (Figures S1A and S1C in Text S1). In contrast, CMV-specific CD8^+^ T cells from SLE patients retain their cytotoxic potential (Figures 3B and 3D lower panels). We conclude from this first set of experiments that there is an EBV-specific CD8^+^ T cell functional defect in SLE patients, the latter cells being impaired in their capacity to secrete multiple effector cytokines and in their cytotoxic granule exocytosis process.

### PD-1 is upregulated on EBV-specific CD8^+^ T cells from SLE patients

**Figure 4:**
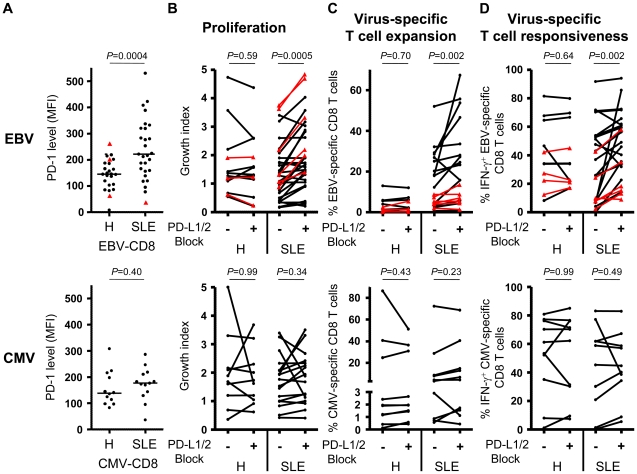
Blockade of PD-1 signalling revigorates EBV-specific T cell responses. Cytofluorometric analysis of PD-1 expression on lytic (black circles) and latent (red triangles) EBV- (upper panel) as well as CMV-specific (lower panel) CD8^+^ T cells. (B) Overall cell growth, (C) virus-specific T cell expansion and (D) IFN-γ secretion by peripheral virus-specific CD8^+^ T cells from healthy controls (H) and SLE patients (SLE) stimulated for 10 days with EBV cognate antigen in the presence (+) or absence (−) of PD-L1 and PD-L2 antagonistic antibodies. Statistical comparisons are performed using (A) Mann-Whitney and (B–D) Wilcoxon matched pairs test.

To investigate the mechanism of EBV-specific CD8^+^ T cell dysfunction, we performed a comparative combinatorial analysis of markers expressed by SLE versus control CD8^+^ T cells. We measured expression levels of a range of differentiation (CD45RA, CCR7, CD27, CD57, FoxP3), co-stimulatory/co-inhibitory (CTLA-4, ICOS, PD-1, CD80, CD86, 41BBL, ICOSL and PD-L1), activation (HLA-DR, CD69 and CD38) and proliferation (Ki-67) markers on EBV-specific cells and total CD8^+^ T cells. We found that the balance between central memory, effector memory and naïve CD8^+^ T cell subsets is not altered in SLE patients, compared to healthy controls (data not shown). However, proliferation (Ki-67) and activation (HLA-DR, CD69 and CD38) markers are significantly up-regulated on total CD8^+^ T cells in active SLE patients and less pronounced in inactive SLE patients compared to controls (Figures S3A–B in Text S1). Also EBV-specific T cells show a trend to be more activated in SLE patients compared to healthy controls (Figure S3C in Text S1). In addition, we found that whereas inhibitory receptor CTLA-4 expression is conserved (Figure S4A in Text S1), PD-1 expression is up-regulated on total CD8^+^ T cells (p = 0.005 and 0.008 for inactive and active SLE, respectively) compared to healthy controls (Figure S4B in Text S1). Interestingly, polyclonal stimulation of CD8^+^ T cells with Staphylococcal Enterotoxin B (Figure S4C in Text S1), anti-CD3 and anti-CD28 antibodies (Figure S4D in Text S1) or PMA-Ionomycin (Figure S4E in Text S1) mounted lower responses in SLE patients compared to healthy controls. Importantly, EBV-specific CD8^+^ T cells represent one of the T cell subsets expressing high PD-1 levels in SLE, compared to controls (Figure 4A; p = 0.0004). In contrast, CMV-specific CD8^+^ T cells from SLE patients do not express elevated levels of PD-1 (Figure 4A).

### PD-1 signaling constrains EBV-specific CD8^+^ T cells from SLE patients

Since PD-1 expression has previously been associated with impaired cellular functionality,[22] we then asked whether increased PD-1 expression by EBV-specific CD8^+^ T cells from SLE patients could account for their impaired functional capacity. In HIV-infected patients, it was shown that blockade of the PD-1 inhibitory pathway can restore CD8^+^ T cell functionality.[23] We therefore tested the influence of the PD-1 signaling pathway on EBV-specific CD8^+^ T cells by blocking PD-1 signaling with antagonistic antibodies specific for PD-1's two known ligands, PD-L1 and PD-L2. Blockade of PD-1 signaling during lytic and latent EBV antigen stimulation substantially boosted general T cell proliferation (Figure 4B), EBV-specific T cell expansion (Figure 4C) and IFN-γ secretion (Figure 4D) in PBMC cultures from SLE patients but not from healthy controls. In contrast, blockade of PD-1 signalling during CMV antigen stimulation neither boosted general T cell proliferation (Figure 4B) nor CMV-specific T cell expansion (Figure 4C) or IFN-γ secretion (Figure 4D). We conclude that the PD-1 inhibitory pathway appears to have a particularly important deleterious impact on lytic and latent EBV-specific CD8^+^ T cell responses in SLE patients.

### EBV replication peaks post initiation of SLE disease flare

**Figure 5:**
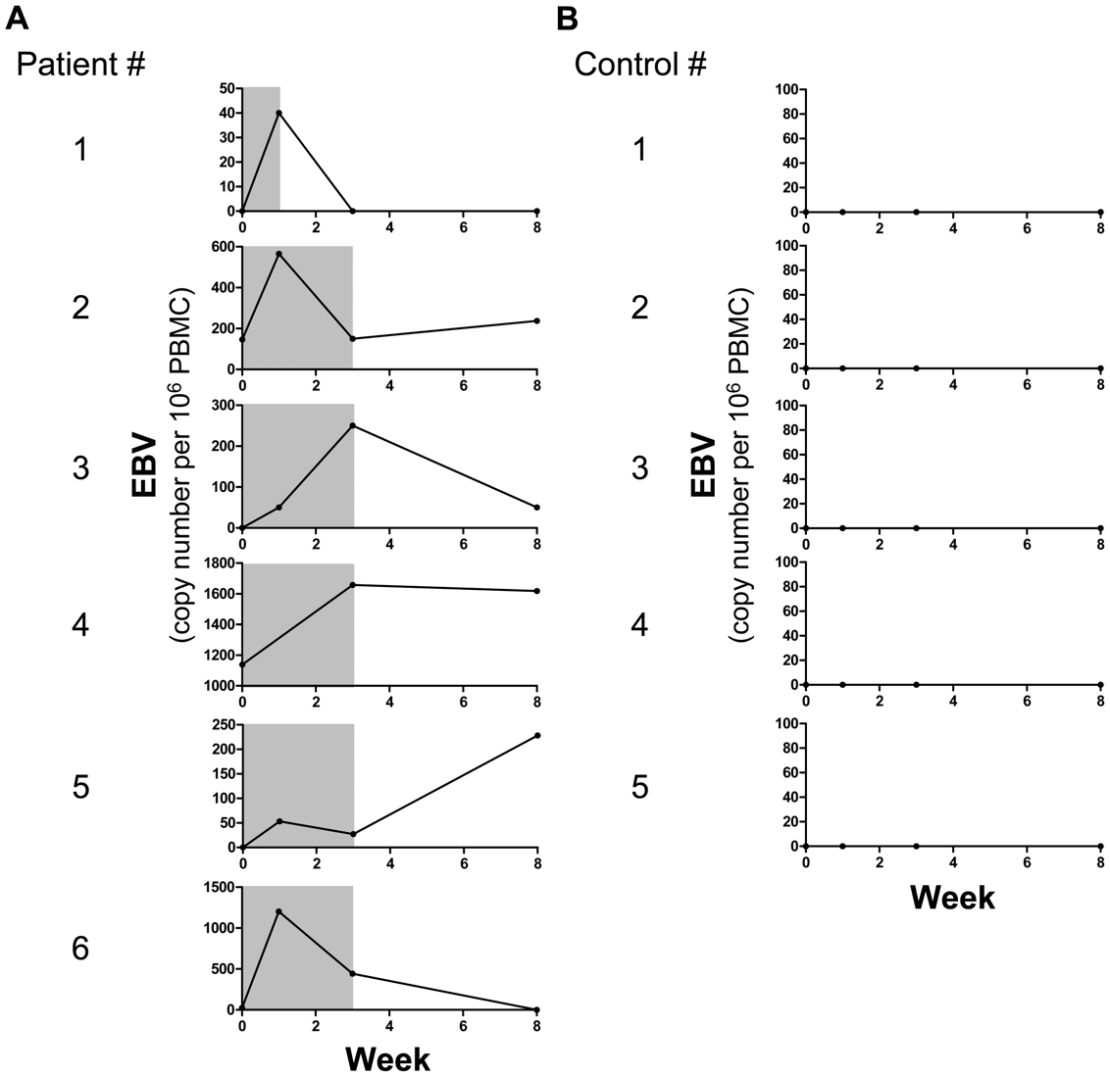
Longitudinal monitoring of EBV replication following SLE flare onset. EBV viral load as genome copies per 10^6^ PBMCs (black line) and synchronous disease activity (gray shading, SLEDAI≥6) in (A) 6 SLE patients and (B) 5 healthy controls.

Although EBV replication was found increased both in active and inactive patients, we reasoned that only longitudinal studies would clearly decipher whether EBV viral bursts precede or follow disease flares. In order to address this issue, SLEDAI and EBV viral load were longitudinally recorded from initiation of disease flare to clinical and biological recovery in 6 established SLE patients (Figure 5A) and 5 healthy controls (Figure 5B). An increase of EBV viral load was observed in all SLE patients (Figure 5A). In contrast, EBV remained below detection levels in the 5 healthy controls monitored during the 8 weeks follow-up (Figure 5B). Importantly, viral replication peaked 1 week or more post flare onset in all 6 patients followed longitudinally, EBV being below detection level in 4 of these patients at time of hospital admission (Figure 5A). We confirmed in the cross-sectional series of flaring patients that EBV was below detection levels in 5 out of 7 cases studied at the time of their hospital admission. We conclude from these cross-sectional and longitudinal studies that early clinical symptoms of SLE do not coincide with high EBV viral load.

## Discussion

Alterations in the control of EBV infection in individuals susceptible to lupus are suspected to promote the development of autoimmunity through multiple mechanisms, such as cross-reactive antibody and T cell responses.[24] Here we show that SLE patients have recurrent bursts of EBV viral load. We furthermore associate this altered control of EBV infection with a PD-1 induced impairment of T cell mediated immune surveillance of EBV.

Virus-specific T cells play a crucial role in the control of EBV infection, and have already been the focus of previous studies in human SLE.[12], [19] Berner *et al.* addressed the issue by combining MHC-peptide tetramer staining with IFN-γ ELISPOT analysis. Based on these tests, it was suggested that EBV-specific T cells from SLE patients might have impaired IFN-γ secreting capacity.[20] The latter study was however hampered by limitations in cohort size, and by the fact that function and frequency of EBV-specific CD8^+^ T cells were not monitored simultaneously at the single cell level.

The present study was designed to concurrently assess the quality and quantity of EBV-specific CD8^+^ T cell responses. This was achieved by combining the analysis of IFN-γ, TNF-α IL-2, MIP-1β, CD107a and granzyme B on MHC class I tetramer-stained EBV-specific CD8^+^ T cells stimulated with their cognate antigen. Being able to enumerate not only frequencies of responses, but also proportions of functional cells among EBV-specific CD8^+^ T cells, we clearly establish that EBV-specific CD8^+^ T cells are present at slightly elevated frequency but functionally impaired in SLE patients. Indeed, EBV-specific T cells from SLE patients exhibit a reduced capacity to secrete IFN-γ, TNF-α, IL-2 and MIP-1β and an impaired cytotoxic granule exocytosis process. The increased frequency of CD8^+^ T cells specific for lytic EBV antigens is most likely due to recurrent EBV replication. However, the elevated frequency is counterbalanced by a global T cell lymphopenia, which is a common clinical feature of SLE.[25] Furthermore, functional impairment at the single-cell level coincides with a diminished absolute number of functional EBV-specific CD8^+^ T cells in SLE patients. Interestingly, there was no direct inverse correlation between EBV-specific cell function (cytokine secretion and cytotoxicity) and EBV viral load (data not shown). This is probably related to the fact that EBV viral loads fluctuate relatively rapidly (Figure 5) and frequently enough to have a long lasting imprint on T cell functions.

A link between CMV and SLE has also been debated due to the fact that more frequent CMV seropositivity and elevated CMV viral loads have been reported in SLE patients in a single study.[26] SLE patients from the present study were also found more frequently seropositive for CMV than healthy controls (Table 1). However, CMV viral loads were not found elevated and dysfunctional anti-CMV T cell responses were not observed in SLE patients, compared to healthy controls. Altogether, the immune alterations described in our study affect preferentially EBV-specific responses and not responses to another herpesvirus, CMV.

The impaired functional status of EBV-specific T cells in SLE patients could be due to an alteration in their phenotype, possibly caused by recurrent exposure to EBV antigens. We observed (Figure S3 in Text S1) that proliferation marker Ki-67 and activation markers CD69, HLA-DR and CD38 were up-regulated on CD8^+^ T cells from SLE patients as previously reported.[20], [27], [28] Taken together, this demonstrates that T cell hyper activation and hyper proliferation are essential factors in SLE pathophysiology.

PD-1 has previously been associated with diminished functional capacity [22] and up-regulation is commonly observed on chronically stimulated antiviral T cells.[23], [29] Of note, a single nuclear polymorphism (SNP) within the gene encoding the PD-1 receptor has been identified as an inheritable risk factor of SLE.[30] We therefore reasoned that the PD-1 receptor could be involved in the EBV-related immune alterations observed in SLE patients. As shown, compared to control lytic EBV-specific CD8^+^ T cells, PD-1 surface expression levels are indeed up-regulated on lytic EBV-specific CD8^+^ T cells from SLE patients. The functional relevance of this marker was corroborated by the fact that blocking PD-1 signaling restores both lytic and latent EBV-specific CD8^+^ T cell function.

PD-1 expression is not only up-regulated on EBV-specific CD8^+^ T cells but also, most likely, on pathogenic T cells, since elevated PD-1 levels are observed on the global CD8^+^ T cell compartments (Figure S4B in Text S1). We also observed that not only EBV-specific T cells show signs of impairment in SLE patients as polyclonal stimulation reveal significantly diminished cytokine responses in the global CD8^+^ T cell compartment (Figures S4C-E in Text S1). Therefore PD-1 up-regulation in SLE patients might represent an important regulatory mechanism, limiting the severity of pathogenic T cell responses. This view is also supported by the fact that a recessive PD-1 knock-out SNP is overrepresented in families of individuals suffering from SLE,[30] suggesting a protective role for PD-1 regulation in SLE immunopathogenesis.

It is still debated whether EBV reactivation is a cause or consequence of SLE disease activity. We first noted that EBV replication in our initial cross-sectional studies is usually undetectable at time of hospital admission for SLE flare (5 out of 7 cases). To address this issue more directly we longitudinally followed patients starting at their first hospital visit after initiation of disease flare until flare resolution. In this way we observed that EBV replication is maximal post flare onset. The relatively narrow window of EBV replication assessed through longitudinal analysis suggests that cross-sectional studies most probably underestimate the occurrence of EBV reactivation in active patients. This would explain why no significant differences were recorded between active and inactive patients in terms of EBV viral loads (Figure 1). More longitudinal studies will be necessary to formally rule out the implication of EBV in the triggering of SLE flares. In particular, it would be interesting to monitor EBV not only at flare onset, but also shortly before active disease. Nevertheless our results strongly suggests that EBV replication is more likely a result of B cell activation associated with active disease, rather than a triggering factor for disease re-activation.

However, EBV can contribute to the vicious circle of autoimmunity in several ways. As previously mentioned, EBV can be responsible for the induction of cross-reactive B and T cell responses.[15], [16] Moreover, it was shown in healthy individuals that EBV induces type 1 interferon (IFN) production by plasmacytoid dendritic cells,[31] a subset of cytokines which are central features of SLE active disease.[32] Thus, iterative episodes of viral replication could account, at least in part, for the over-expression of IFN and IFN-induced genes observed in SLE.[33], [34] The potential implications of EBV in SLE immunopathology in relation to an impaired EBV-specific T cell response suggest that pharmaceutical or immunological anti-EBV interventions might potentially be beneficial to these patients.

In conclusion we propose a model where autoimmune-driven B cell activation [35], [36] induces an activation of the EBV lytic cycle in infected B cells, which leads to a burst of EBV replication. In response, EBV-specific T cells are activated in order to control viral replication and may eventually cross-react with self antigens and lead to auto-immune manifestations. EBV-induced IFN may also take part in SLE immunopathology. Repetitive episodes of viral replication ultimately results in PD-1 mediated impairment of EBV-specific cytotoxic and cytokine-secreting T cells. This impairment partially limits the risks of cross-reactive tissue injuries, but at the same time explains why EBV replication is less suppressed in SLE patients.

Association between SLE and EBV has been studied for 40 years, and EBV remains suspected to induce SLE early on in life.[37], [38] In established SLE disease, it is debated whether autoimmunity is triggered by reactivation of pathogens, such as EBV or *vice versa*.[2] In our study of adults with established disease, frequent EBV reactivation appears to be an aggravating consequence, rather than a cause, of SLE immunopathology. Future studies are needed to elucidate whether EBV contributes to the initiation of disease in young healthy individuals.

## Materials and Methods

### Ethics statement

All samples were obtained following acquisition of the study participants' and/or their legal guardians' written informed consent. The study protocol was reviewed and approved by the local ethics committees (Comité de Protection des Personnes Ile de France VI).

### Patients and healthy donors

We enrolled a total of 149 study subjects, including 118 consecutive SLE patients, defined according to the American College of Rheumatology classification criteria,[39] as well as 31 healthy (H) control subjects. SLEDAI for individual SLE patients was determined at the time of sample collection.[40] SLE patients were subdivided in two groups consisting of 76 inactive (SLEDAI<6) and 42 active (SLEDAI≥6) SLE patients. Included subjects were then selected according to their HLA genotype (HLA-A*0201, A*1101, B*0702, B*0801), for which well characterized EBV and CMV peptide antigens have been described.[41], [42], [43], [44]

### EBV and CMV serology and quantification

The serological status of EBV and CMV were measured by serum ELISA (BIO Advance, France) according to the manufacturer's instructions. Both EBV and CMV DNA loads were measured using in-house real-time PCR assays. EBV and CMV PCRs were carried out on the same DNA extract obtained from peripheral blood mononuclear cells (PBMCs) or total blood for longitudinal studies, using the QIamp Blood DNA kit (Qiagen, France) according to the manufacturer's instructions. Real-time quantitative PCRs based on hydrolysis probe technology were carried out on a LightCycler 480 (Roche Diagnostics, France) as previously described by Deback *et al.* [45] Real-time PCR accuracy was previously confirmed by the Quality Control for Molecular Diagnosis (QCMD) 2008 proficiency panel. The human albumin gene was quantified in each DNA sample, to enable quantitation of the copy number per million cells of EBV and CMV.

### Antibodies and peptide/MHC tetramers

Directly conjugated and unconjugated antibodies were obtained from the following providers: BD Biosciences (San Jose, CA): Ki-67 [FITC], HLA-DR [PE–cyanin 7], CD38 [Alexa Fluor 700], CTLA-4 [cyanin 5-PE], CD107a [cyanin 5–PE], Granzyme B [A647], IFN-γ [Alexa Fluor 700], IL-2 [APC] and TNF-α [PE–cyanin 7]; R&D Systems (Abingdon, UK): MIP-1β [FITC], PD-1 [FITC]; Caltag (Burlingam, CA): CD8 [Alexa Fluor 405]; Dako (Glostrup, Denmark): CD3 [cascade yellow] and BioLegend (San Diego, CA): CD69 [APC-Cy7]. Peptide/MHC tetramers were produced as previously described [41] and included the following epitopes: HLA-A*0201 CMV pp65-NV9; HLA-A*0201 EBV BMLF1-GL9 and BMRF1-YV9; HLA-A*1101 EBV EBNA-3B IK9, EBNA-3B AK10; HLA-B*0702 CMV pp65-TM10; HLA-B*0801 EBV BZLF1-RL8 and EBNA-3A-FL9.

### Cytometry and polyfunctional analysis

PBMCs isolated on ficoll gradients (PAA, France) were stained with titrated antibodies specific for cell surface markers, followed by staining for intra-cellular Ki-67, according to manufacturer's recommendation.

For polyfunctional analysis, PBMCs were stimulated in the presence of peptide antigen (5 µM) and PE-Cy5 conjugated anti-CD107a antibody over night at 37°C in a 5% CO_2_ incubator. Cytokine secretion was blocked by the addition of 2.5 µg/ml monensin and 5 µg/ml Brefeldin A (Sigma-Aldrich, St. Louis, MO). Cells were then stained with corresponding PE-conjugated peptide MHC class I tetramer (0.5 µg per 10^6^ cells) and directly conjugated anti-CD3 and anti-CD8 antibodies. Cells were then fixed and permeabilized with Cytofix/Cytoperm (BD Biosciences) according to manufacturer's instructions. Finally, cells were stained with anti-cytokine antibodies and/or anti-granzyme B antibody for 15 minutes at room temperature.

Samples were acquired on a BD LSRII flow cytometer (Becton Dickinson) with appropriate isotype controls and color compensation. Data were analysed with FACSDiva (BD Biosciences) and FlowJo (TreeStar Inc) softwares. Unstimulated cells for each sample, treated under the same experimental conditions served as negative controls, and background values were subtracted from the analysis of the stimulated samples.

### Blockade of PD-1 signal pathway

PBMCs were cultured for 10 days at 37°C 5% C02, in RPMI supplemented with 5% human serum and a cytokine cocktail mix (20 ng/ml of IL-7 and 20 ng/ml IL-2 (R&D Systems, Minneapolis, MN)). Cells were stimulated with or without EBV or CMV peptide (1 µg/ml) in the presence of either isotype control antibodies or both anti-PD-L1 and anti-PD-L2 (10 µg/ml). On day 10, cells were re-stimulated with peptide (1 µg/ml) overnight and proliferation and functionality was assessed by cell counting and flow cytometry. Antagonistic antibodies were kindly provided by Pr. Gordon Freeman (Dana Farber Institute, Boston).

### Data management and statistical analysis

Clinical information and flow cytometric analysis were gathered in a database (Office Access 2003, Microsoft France, Issy-les-Moulineaux, France).

Differences of continuous variables between patient groups were tested using the Mann-Whitney U-test (unpaired) and the Wilcoxon matched pairs test (paired). Differences of categorical variables, such as sex and detectable viral load, between groups were tested with Fisher's exact test. All tests were 2-sided and a p value <0.05 was considered statistically significant. To exclude the influence of treatment-related factors on EBV viral load and EBV specific CD8^+^ T cell cytokine secretion we built multivariate regression models. In these models, EBV viral load and EBV specific CD8^+^ T cell cytokine secretion were used as dependent variables, and all treatment-related variables were included as explanatory variables. Statistical analysis was performed using GraphPad Prism Ver. 4.03 (GraphPad Software Inc), JMP7 (SAS Software, NC, USA), Pestle Ver. 1.6.2 and Spice Ver. 4.2.3 (Mario Roederer, ImmunoTechnology Section, VRC/NIAID/NIH) softwares.[46]

## Supporting Information

Text S1 Supplemental Materials and Methods and 4 supplemental figures.
(PDF)
