# Supplementary material for: Exhausted Cytotoxic Control of Epstein-Barr Virus in Human Lupus
Source: PLoS Pathog. 2011 Oct 20;7(10):e1002328. doi: 10.1371/journal.ppat.1002328 (PMC3197610; doi:10.1371/journal.ppat.1002328)

Supplemental Materials and Methods

Functional analysis of polyclonally stimulated T cells

For polyfunctional analysis, PBMCs were stimulated in the presence of 1) 2µg/ml Staphylococcal Enterotoxin B (SEB) (Sigma-Aldrich), 2) 10µg/ml bound anti-CD3 (OKT3, Orthoclone; Orthobiotech) and 10µg/ml soluble anti-CD28 (BD Biosciences) antibodies and 3) 1µg/ml PMA and 1µg/ml Ionomycin (Sigma-Aldrich). The remaining experimental procedure is equivalent to the procedure outlined in the main manuscript.

Supplemental figure legends

Figure S1

Multiparametric functional assessment of latent EBV-specific CD8$^+$ T cells. (A) Representative cytofluorometric detection (left) and functional analysis (right) of CD8$^+$ T cells specific for the latent EBV antigen EBNA3A in a healthy control (upper panel) and in an inactive SLE patient (lover panel) post peptide antigen stimulation of PBMC. EBV latent antigen-specific cells were detected with peptide/MHC tetramer and anti-CD8 (red box) and simultaneously analyzed for intra-cellular IFN-γ content and CD107a surface expression. Gates were positioned according to control stains of non-stimulated virus-specific T cells. (B) Magnitude and (C) functionality of EBV-specific responses in healthy controls (H, n=4) and SLE patients (S, n=4). P-values monitoring differences between healthy donors and SLE patients are calculated using a non-parametric Mann-Whitney test.

Figure S2

Reduced absolute numbers of functional lytic EBV- and CMV-specific CD8$^+$ T cells in SLE patients. (A) Absolute CD4$^+$ and CD8$^+$ T cell counts in whole blood reveal an extensive (~2 fold) lymphopenia in SLE patients. (B) Absolute and (C) functional numbers of EBV lytic antigen- (upper panel) and CMV antigen-specific (lower panel) CD8$^+$ T cells in healthy controls (H, n=29 and 15, respectively), inactive (i, n=12 and 8, respectively) and active (a, n=17 and 7, respectively) SLE patients (see figure 2 for more details). P-values monitoring differences between healthy donors and SLE patients are calculated using a non-parametric Mann-Whitney test.

Figure S3

Proliferation, inhibitory and activation markers on $CD8^+$ T cells. *Ex-vivo* frequency of (A) Ki-67 and (B) CD69, HLA-DR, CD38 expressing $CD8^+$ T cells as well as (D) CD38 expressing EBV-specific $CD8^+$ T cells. Group comparison between healthy controls (H), inactive (i) and active (a) SLE patients (SLE) were performed using a non-parametric Mann-Whitney test.

Figure S4

Phenotypic and functional assessment of $CD8^+$ T cells from SLE patients. Cytofluorometric analysis of inhibitory receptors (A) CTLA-4 and (B) PD-1 expressions on $CD8^+$ T cells. (C) Peripheral T cells were stimulated with Staphylococcal Enterotoxin B (SEB), (D) anti-CD3 and anti-CD28 antibodies and (E) PMA-Ionomycin. Subsequently, $CD8^+$ T cells were analyzed for intra-cellular IFN-γ and TNF-α content. Cytokine gates were positioned according to control stains of non-stimulated T cells. Frequency of functional T cells towards SEB, anti-CD3+anti-CD28 and PMA-Ionomycin in healthy controls (H, n=9, 32 and 22, respectively) and SLE patients (SLE, n=9, 16 and 17) were compared using a non-parametric Mann-Whitney test.

Supplemental Figure S1

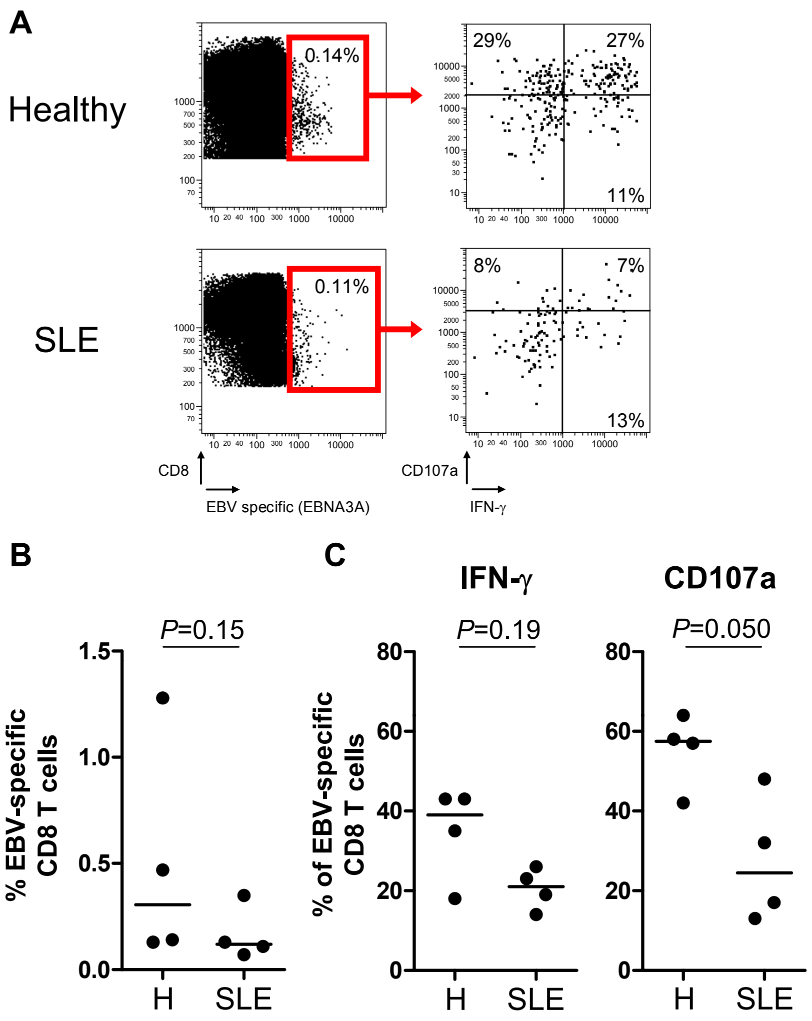

Supplemental Figure S2

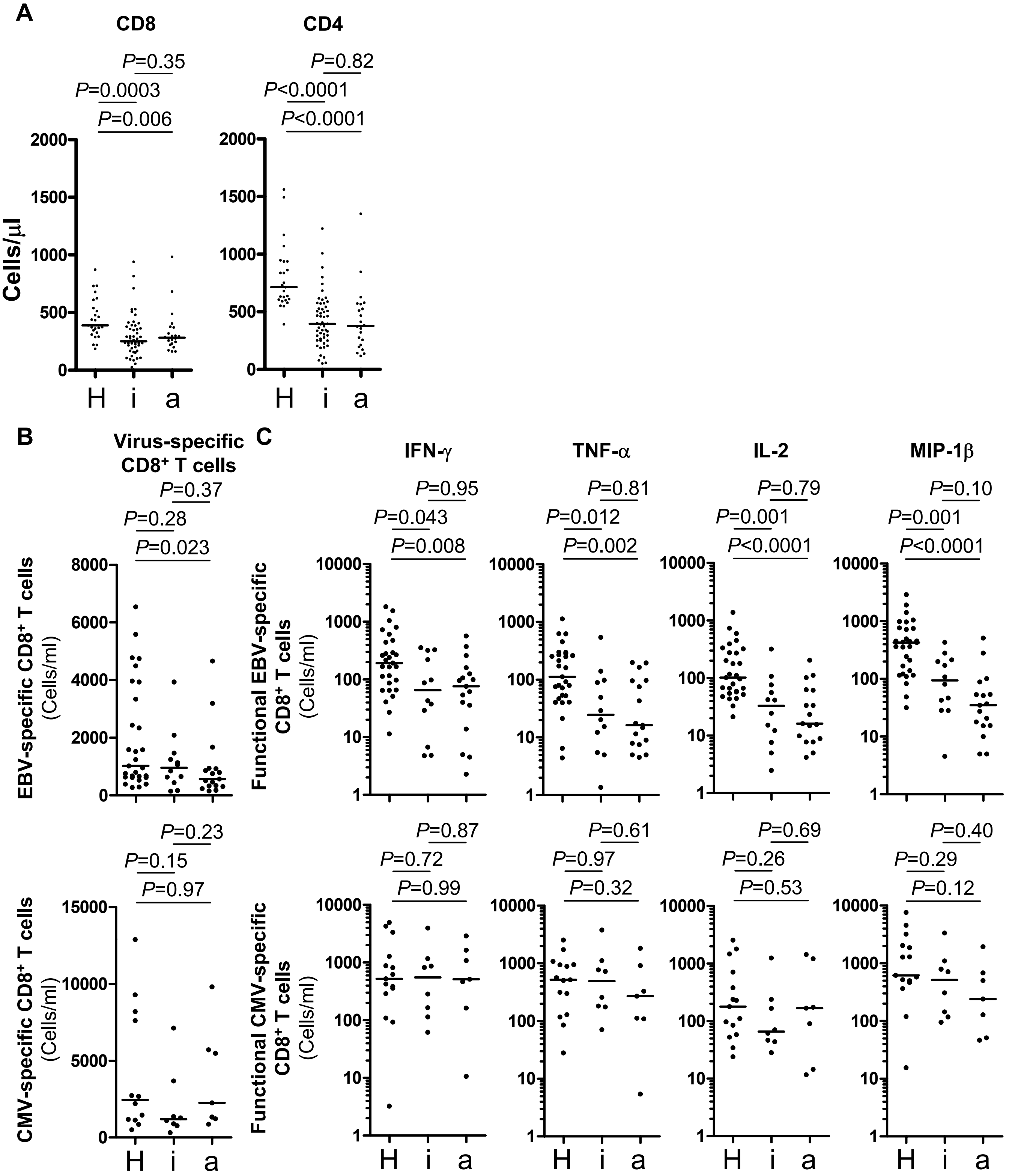

Supplemental Figure S3

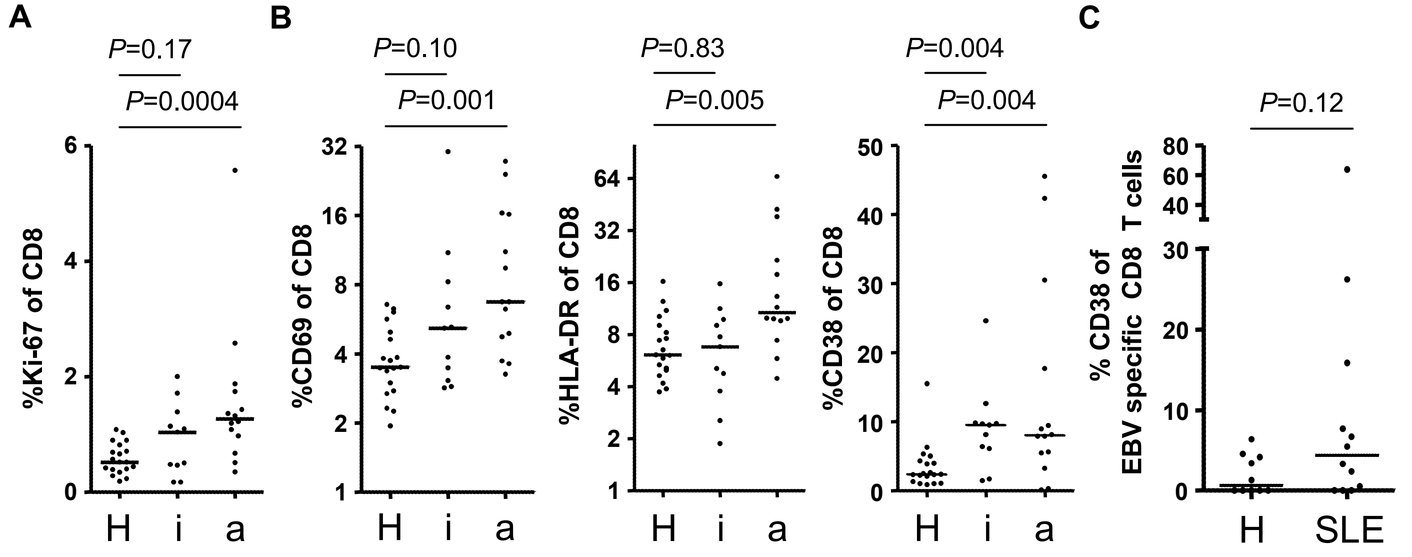

Supplemental Figure S4

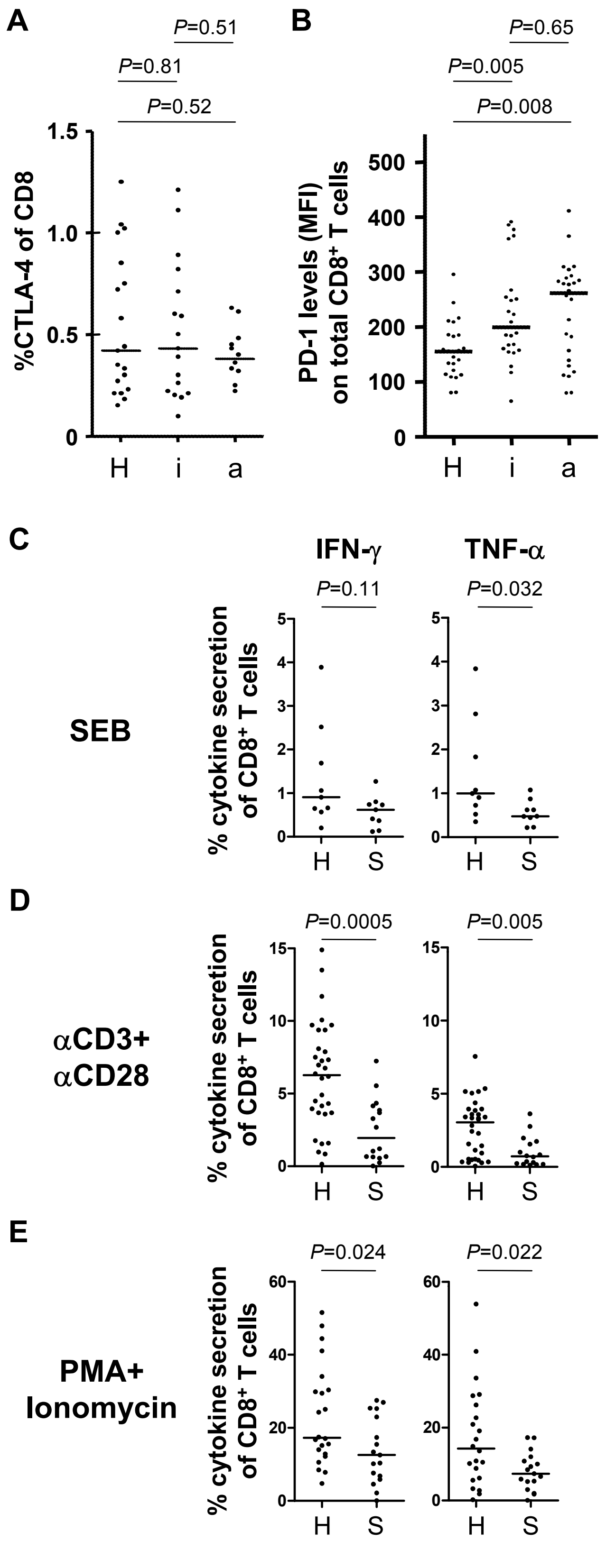

Supplement: Text S1 — Supplemental Materials and Methods and 4 supplemental figures. (PDF) [file ppat.1002328.s001.pdf]
